# The Influence of Fe_2_O_3_ Nanoparticles on the Thermal Degradation and Kinetics of PMMA

**DOI:** 10.3390/polym18070790

**Published:** 2026-03-25

**Authors:** Aytekin Ulutaş, Mesut Eryiğit

**Affiliations:** 1 College of Edremit Civil Aviation, Balıkesir University, 10300 Balıkesir, Türkiye; 2 Chemistry Department, Faculty of Science and Art, Balıkesir University, 10145 Balıkesir, Türkiye; mesut.eryigit@balikesir.edu.tr

**Keywords:** poly(methyl methacrylate), Fe_2_O_3_ nanoparticles, thermal degradation, degradation kinetics, nanocomposites, activation energy

## Abstract

Fe_2_O_3_-reinforced PMMA nanocomposites were prepared by melt blending using a twin-screw micro-extruder. Fixed Fe_2_O_3_ loading of 2.5 wt.% was employed, and mixing times of 6 and 12 min were used to obtain nanocomposites with different dispersion characteristics. The structural and morphological properties of the samples were investigated by X-ray diffraction (XRD) and scanning electron microscopy (SEM), while their thermal degradation behavior was evaluated by differential thermal and thermogravimetric analyses (DTA/TG). The activation energies of thermal degradation were calculated using the Kissinger, Takhor, and Augis–Bennett methods. Increasing the mixing time improved nanoparticle dispersion and reduced agglomeration. The addition of Fe_2_O_3_ slightly decreased the characteristic degradation temperatures of PMMA, while the activation energy increased for the better-dispersed sample. The results indicate that interfacial interactions and particle dispersion play important roles in the thermal degradation behavior of PMMA/Fe_2_O_3_ nanocomposites.

## 1. Introduction

Hybrid polymer nanocomposites containing various inorganic fillers or additives have received great attention due to their advanced mechanical, thermal, and electrical properties; lower cost; ease of molding; lightness; and potential in a wide range of technological applications [1,2,3,4,5,6]. Polymer nanocomposites with high thermal conductivity and a high dielectric constant are increasingly required for various applications in the electrical and electronics industries, such as voltage control systems, sensors, actuators, embedded capacitors, electromagnetic shielding, and emerging energy-storage devices [7]. Therefore, issues such as heat removal and mechanical stability are of prime importance and must be improved in order to maximize the performance of various polymers in the electronics industry [8]. In this context, poly(methyl methacrylate) (PMMA) is an important engineering polymer because of its transparency, low density, and ease of processing; however, its thermal degradation behavior at elevated temperatures limits its reliability in applications requiring thermal resistance. Therefore, understanding and controlling the degradation behavior of PMMA is essential for improving its practical performance.

The incorporation of nanoparticles in a polymer matrix alters, as expected, the mechanical, thermal, and other characteristics of the polymer [9]. In PMMA-based systems, metal oxide nanoparticles may affect degradation behavior through interfacial interactions with polymer chains, by introducing heterogeneous regions in the matrix, and by modifying heat transfer during thermal treatment. Depending on the dispersion state, these effects may either delay or facilitate the onset of thermal decomposition. Rocha et al. investigated the thermal properties of maghemite/poly(methyl methacrylate) (PMMA) nanocomposites. They reported that increasing the maghemite content also increased the thermal stability of the nanocomposite compared to pure PMMA. They also showed that the electron density within the maghemite nanoparticles was not homogeneous and that low-electron-density regions acted as radical scavengers during PMMA degradation, thereby contributing to thermal stabilization [10]. Dallas et al. prepared polymer/magnetic nanoparticle composites in their study and investigated the thermal properties of the obtained nanocomposites. They reported that an increase in the glass transition temperature was observed after the addition of nanoparticles [11]. Poly(methyl methacrylate) (PMMA) is a transparent thermoplastic polymer, known for its high optical transparency, low density, resistance to weather conditions, and ease of processing [1,5]. Compared with many other polymers, PMMA is particularly distinguished by its high transparency and relatively stable chemical structure. PMMA is synthesized from the methyl methacrylate monomer (C_5_H_8_O_2_), and the repeating unit of the polymer chain is expressed as [–CH_2_–C(CH_3_)(COOCH_3_)–]_n_. The ester groups present in the PMMA chain structure can promote interfacial interactions with Fe_2_O_3_ nanoparticles; therefore, PMMA is considered a suitable polymer matrix for nanocomposite formation [3,6]. Fe_2_O_3_ nanoparticles were selected due to their chemical stability, low cost, and good compatibility with polymer matrices. Previous studies have shown that Fe_2_O_3_ can influence the thermal stability and degradation behavior of polymer nanocomposites [3,10,11].

The polymorphic nature of iron oxides is well known. Iron oxides and their nanoparticles are ubiquitous in the environment and have been investigated due to their properties and functions in both natural and engineered systems [12]. Laachachi et al. investigated the thermal properties of PMMA composites containing TiO_2_ and Fe_2_O_3_ nanoparticles at loadings of 5%, 10%, 15%, and 20% by weight, and they observed an increase in thermal stability with increasing oxide content [13]. Barandiaran et al. modified Fe_2_O_3_ magnetic nanoparticles with APTS silane in order to improve their dispersion, and they reported that the resulting PMMA nanocomposite exhibited a more homogeneous structure [14]. Aymonier et al. reported that the degradation of a PMMA composite containing 0.0005 vol.% Pd was delayed by more than 75 °C, while its optical and thermal properties were preserved [15]. These studies clearly show that interactions between polymers and additives can play a critical role in determining the properties of nanocomposites [5]. In particular, these studies suggest that Fe_2_O_3_ nanoparticles can influence PMMA not only through filler addition itself but also through the quality of particle dispersion, which may strongly affect the resulting degradation pathway and thermal response of the nanocomposite.

Heat dissipation during the operation of micro- and nanoelectronic devices remains a major challenge. To control the heat generated in such systems, polymeric materials with improved thermal conductivity are required to dissipate excess heat efficiently. For such applications, the thermal response of PMMA-based materials becomes especially important, since local heating may trigger structural instability and accelerate degradation during service.

In our previous study, the effect of the mixing time on the thermal stability and activation energies of NiO/PMMA nanocomposites was investigated. The addition of NiO nanoparticles facilitated earlier decomposition by lowering both the initial temperature and activation energy compared to pure PMMA. These findings indicate that the incorporation of metal oxide nanoparticles can significantly modify the thermal decomposition behavior and apparent activation energy of PMMA, depending on the oxide type and dispersion state [16].

A crucial challenge in the preparation of nanoscale composites is preventing nanoparticle aggregation [17]. Therefore, the mixing time is important to ensure the distribution of particles in the matrix [18]. Another important parameter in nanoparticle-reinforced composites is the filler loading. For example, Sun et al. investigated the mechanical behavior of epoxy-reinforced nanoparticles of 4% Fe_2_O_3_ [19]. Naguib et al. investigated the effect of 3% Fe_2_O_3_-coated particles on the mechanical properties of the epoxy matrix [20]. Maleki et al. produced 5%, 10%, 15%, and 20% Fe_2_O_3_ reinforcement particles and polymer matrix composites [21]. Low nanoparticle loadings are commonly preferred in PMMA-based nanocomposites to improve dispersion while minimizing severe agglomeration. Based on literature reports on oxide-reinforced polymer systems, Fe_2_O_3_ loading of 2.5 wt.% was selected in the present study as a representative low loading level sufficient to reveal the effect of the mixing time on the morphology and thermal degradation behavior. At this loading level, the effects of processing-induced dispersion differences can be observed more clearly, without the strong masking effect of excessive particle crowding.

Different mixing times were applied during the preparation of the samples with the selected reinforcement ratio in order to obtain different dispersion states. The main objective of this study was to examine how the mixing time affects the dispersion of Fe_2_O_3_ nanoparticles in PMMA and how this dispersion influences thermal degradation behavior and kinetic parameters. For this purpose, PMMA/Fe_2_O_3_ nanocomposites containing 2.5 wt.% Fe_2_O_3_ were prepared at two different mixing times (6 and 12 min), and their structural, morphological, and thermal properties were comparatively evaluated by XRD, SEM, DTA, and TG analyses. Thermograms recorded at different heating rates were analyzed using the Kissinger, Takhor, and Augis–Bennett methods to determine the apparent activation energy of degradation.

## 2. Materials and Methods

**Figure 1:**
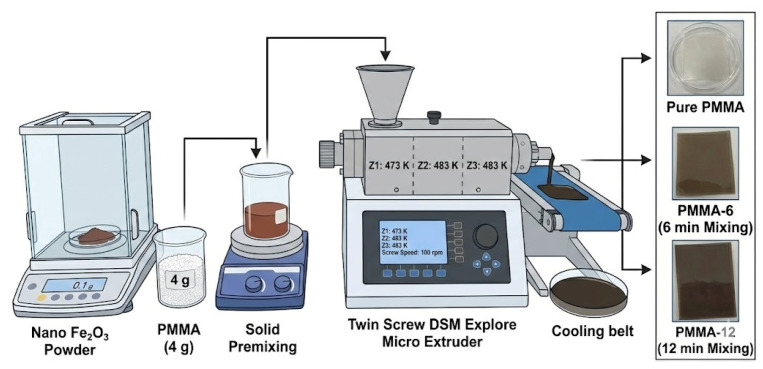
Schematic representation of the synthesis process of pure PMMA and Fe_2_O_3_-reinforced PMMA nanocomposites using the melt-mixing method.

Poly(methyl methacrylate) (PMMA, weight-average molecular weight, Mw ≈ 35,000 g/mol) was purchased from Acros Organics (Geel, Belgium) in powder form. Fe_2_O_3_ nanoparticles (nanopowder, average particle size < 50 nm, Sigma-Aldrich, St. Louis, MO, USA; CAS No. 1309-37-1) were used as the reinforcing phase. All chemical reagents used were of analytical purity. Pure PMMA and Fe_2_O_3_-reinforced PMMA nanocomposites were prepared by melt blending using a twin-screw micro-extruder (DSM Xplore, Sittard, The Netherlands; screw diameter: 5 mm, L/D ratio: 20) [22,23]. PMMA (4 g) and Fe_2_O_3_ nanoparticles (0.1 g) were premixed and fed into the micro-extruder. The processing temperatures were set to 473, 483, and 483 K in the three heating zones, respectively, and the screw speed was maintained at 100 rpm. Two different mixing times, 6 and 12 min, were applied in order to obtain nanocomposites with distinct dispersion states. The resulting samples were designated as PMMA (pure polymer processed under identical conditions), PMMA-6, and PMMA-12, corresponding to mixing durations of 6 and 12 min, respectively. The selected mixing times were chosen to represent two practically distinguishable dispersion conditions while avoiding excessively long residence times, which could induce the thermal degradation of PMMA during melt processing. The schematic flow diagram of the entire experimental procedure, which involved the preparation of pure PMMA and Fe_2_O_3_-doped nanocomposite samples, mixed for different durations using the melt compounding method, is presented in Figure 1.

The structural changes and phases of the samples were determined using a Bruker D8 Advance X-ray diffractometer (Bruker AXS GmbH, Karlsruhe, Germany) with CuKα radiation (λ = 0.154056 nm) over the 2θ range of 5–80° at a step size of 0.013°. For SEM analysis, the samples were fractured and sputter-coated with a thin gold layer prior to imaging in order to improve the electrical conductivity and image quality. The microstructures of the Fe_2_O_3_/PMMA nanocomposites were characterized using a JEOL JSM 6064LV SEM instrument (JEOL Ltd., Tokyo, Japan) at an acceleration voltage of 10 kV. Differential thermal and thermogravimetric analyses were carried out using a Hitachi Exstar SII 7300 instrument (Hitachi High-Tech, Tokyo, Japan). Approximately 10–20 mg of sample was placed in an aluminum pan and heated from room temperature to 1000 K under nitrogen flow [24]. SEM images were obtained at different magnifications in order to evaluate both the overall dispersion and the local agglomeration behavior of Fe_2_O_3_ nanoparticles within the PMMA matrix. The approximate agglomerate sizes were estimated from the SEM micrographs using the scale bars of the corresponding images.

## 3. Results and Discussion

### 3.1. XRD Characterization

**Figure 2:**
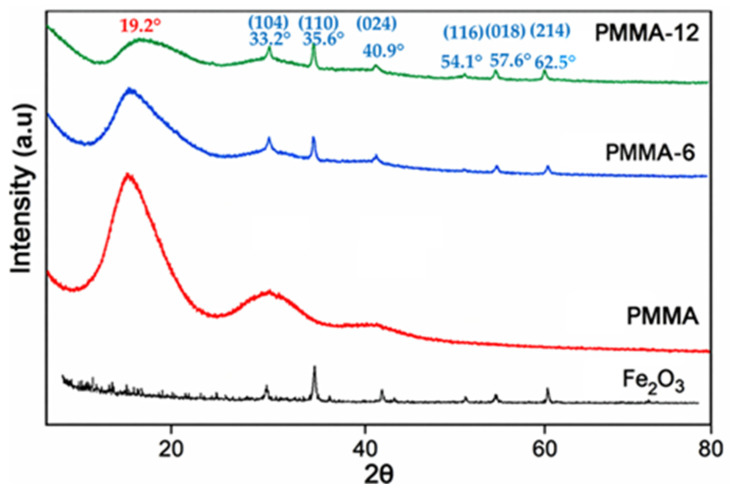
XRD patterns of nano-Fe_2_O_3_, PMMA, PMMA-6, and PMMA-12 samples. The diffraction peaks of Fe_2_O_3_ are indexed according to the hematite crystal structure.

The X-ray pattern of PMMA shows three broad peaks at 2θ = 13.52, 29.61, and 42.22, which are characteristic of the amorphous structure of PMMA. The PMMA reference sample was processed under identical extrusion conditions as the nanocomposites to eliminate processing-related effects and ensure a reliable comparison. The pattern exhibits the typical amorphous nature of the nanocomposite material. The Fe_2_O_3_ loading did not significantly alter the amorphous diffraction profile of PMMA, indicating that the polymer matrix remained predominantly amorphous after melt blending [25]. The XRD results of Fe_2_O_3_ powder and other samples in nanodimensions that were used during the experiments are shown in Figure 2. The XRD patterns of PMMA, PMMA-6, PMMA-12, and Fe_2_O_3_ powder were examined. The patterns of the polymer structure and Fe_2_O_3_ powder combined in nanocomposite PMMA-6 and PMMA-12 samples gave similar peaks at six points at the same angles. The XRD pattern of neat PMMA exhibited broad diffraction halos, characteristic of an amorphous polymer. In the nanocomposite samples, the main diffraction features of Fe_2_O_3_ were also detected, indicating the presence of the oxide phase within the PMMA matrix. The absence of major changes in the PMMA halo suggests that the addition of Fe_2_O_3_ did not induce crystallization in the polymer matrix. Figure 2 shows the XRD pattern for PMMA peaks (19.2°) at 2θ and the relative intensities obtained for the polymer match with the JCPDS Card No. 13-0835 file, identifying it as PMMA. The diffraction peaks observed for Fe_2_O_3_ correspond to the characteristic reflections of the hematite phase. The main peaks, located at approximately 33°, 35°, 49°, 54°, 57°, and 62°, were indexed to the (104), (110), (024), (116), (018), and (214) crystallographic planes, respectively, and are consistent with standard hematite diffraction data (JCPDS Card No. 33-0664, Joint Committee on Powder Diffraction Standards). The presence of Fe_2_O_3_ peaks in the nanocomposite samples confirms the incorporation of the nanoparticles into the PMMA matrix without altering their crystalline structure.

### 3.2. Morphology Characterization

**Figure 3:**
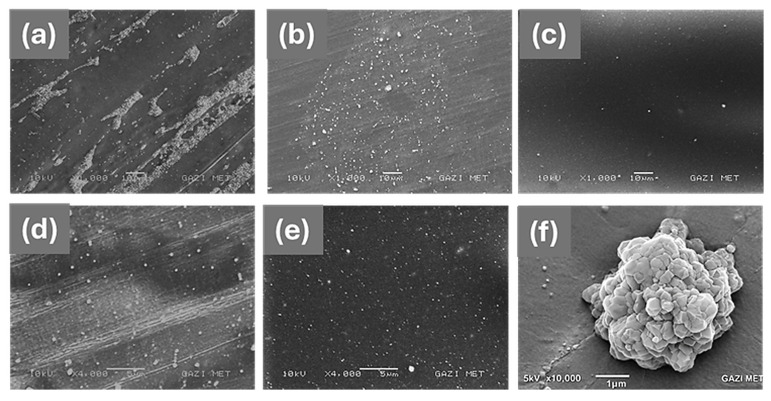
SEM micrographs of pure PMMA and PMMA/Fe_2_O_3_ nanocomposites at different magnifications—(**a**) pure PMMA (1000×), (**b**) PMMA-6 (1000×), (**c**) PMMA-12 (1000×), (**d**) PMMA-6 (4000×), (**e**) PMMA-12 (4000×)—and (**f**) representative detail view of an Fe_2_O_3_ agglomerate in PMMA-12 (10,000×). Bright regions correspond to Fe_2_O_3_ particles/agglomerates dispersed within the PMMA matrix.

The dispersion of Fe_2_O_3_ nanoparticles within the PMMA matrix was examined using scanning electron microscopy (SEM). Figure 3 shows the SEM micrographs of pure PMMA and PMMA/Fe_2_O_3_ nanocomposites obtained at different magnifications in order to evaluate both the overall particle distribution and the local agglomeration behavior. The SEM image of pure PMMA (Figure 3a) exhibits a relatively smooth and homogeneous surface without visible particulate structures. In contrast, bright regions corresponding to Fe_2_O_3_ particles/agglomerates are observed in the Fe_2_O_3_-filled samples (Figure 3b–f). At 1000× magnification, the PMMA-6 sample (Figure 3b) shows a non-uniform particle distribution and visible clustered regions, indicating agglomeration due to insufficient mixing. The PMMA-12 sample (Figure 3c), on the other hand, exhibits a more homogeneous particle distribution with fewer large clustered regions.

The higher-magnification images further support this observation. In the PMMA-6 sample (Figure 3d), the approximate size of Fe_2_O_3_ agglomerates ranges from submicron clusters to several hundred nanometers (approximately 0.15–0.60 µm), with most clusters appearing in the range of approximately 180–320 nm. In contrast, the PMMA-12 sample (Figure 3e) shows significantly smaller agglomerates, ranging approximately between 60 and 240 nm, with the majority of particles distributed in the range of about 90–150 nm. The representative detail view in Figure 3f illustrates the morphology of an Fe_2_O_3_ agglomerate at higher magnification. These observations clearly indicate that increasing the mixing time from 6 to 12 min improves the dispersion of Fe_2_O_3_ nanoparticles and significantly reduces nanoparticle agglomeration within the PMMA matrix. The observed reduction in agglomerate size confirms that the processing conditions play a critical role in controlling nanoparticle dispersion in polymer nanocomposites.

Considering that the primary Fe_2_O_3_ nanoparticle size was below 50 nm, the observed features in the SEM images correspond to secondary agglomerates formed during melt mixing, rather than individual nanoparticles.

### 3.3. Differential Thermal Analysis

**Figure 4:**
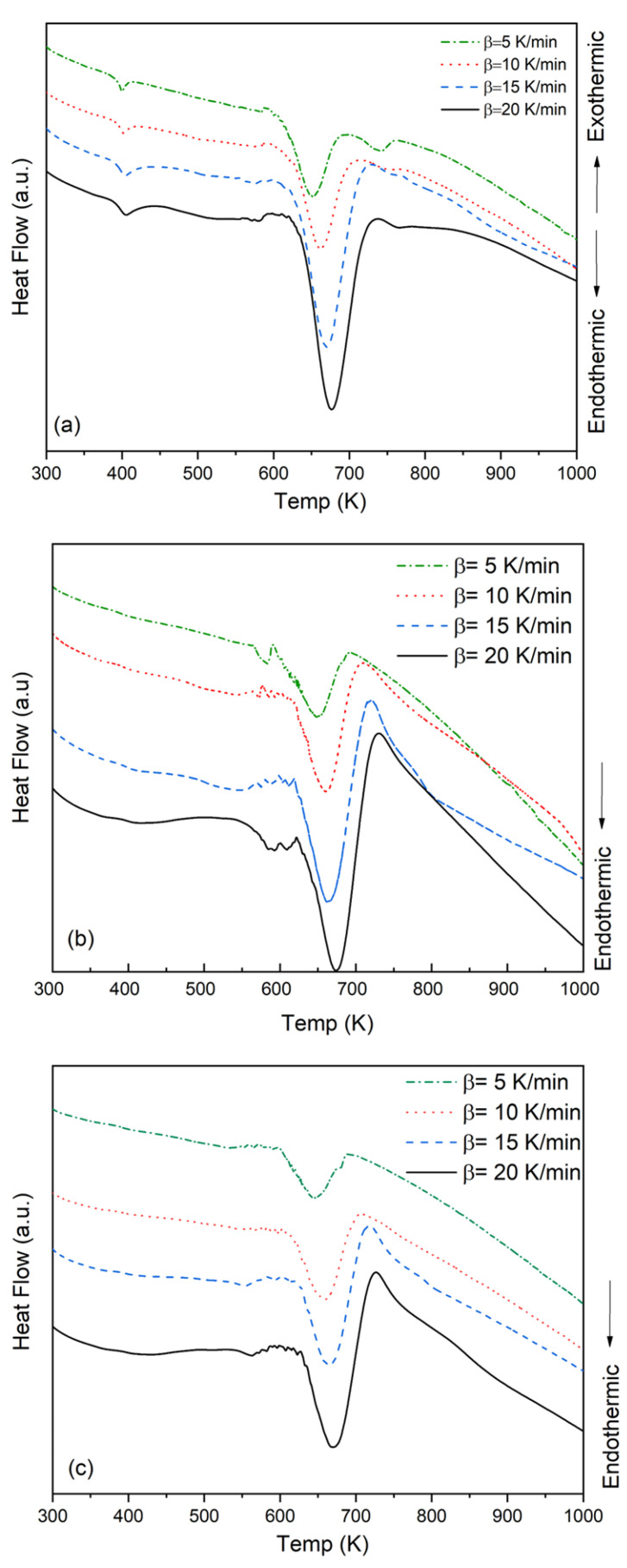
DTA curves of (**a**) PMMA, (**b**) PMMA-6, and (**c**) PMMA-12 samples. The PMMA data are reproduced from our previous study [16] for comparison.

The DTA results demonstrated that the PMMA, PMMA-6, and PMMA-12 nanocomposite samples exhibited endothermic behavior (see Figure 4). As the heating rate increased, the onset temperature of polymer decomposition shifted to higher values due to thermal lag. The pure PMMA sample exhibited a distinct endothermic peak at approximately 680 K. Similar thermal decomposition profiles were observed for the PMMA-6 and PMMA-12 nanocomposites, thus confirming the presence of analogous decomposition mechanisms.

The glass transition temperature of a polymer is important for characterization as it determines the macroscopic performance of the polymer [26]. The glass transition temperature is the temperature at which increased molecular mobility leads to significant changes in the thermal properties of the amorphous structure. The glass transition temperature is an important characteristic parameter for amorphous polymers such as PMMA, as it reflects the onset of enhanced segmental mobility in the polymer chains. It should be distinguished from thermal degradation, which involves chemical bond scission at higher temperatures [6]. Above the glass transition temperature, the polymer tends to expand isotropically [27]. The glass transition temperature (based on the DTA curves given in Figure 4) was found to be about 380 K for pure PMMA. Approximate temperature values are given in the literature [28].

Upon examination of the graph, it was observed that the T_x_ temperatures of the nanocomposite decreased between 3 and 5 K for a constant heating rate, as indicated by the data obtained from DTA. T_x_ is defined as the temperature corresponding to the maximum thermal event peak temperature obtained from the thermal analysis curves. At a temperature increase rate of 5 K/min, the temperatures of the PMMA, PMMA-6, and PMMA-12 samples were recorded as 651 K, 648 K, and 646 K, respectively. The same trend occurred for the other heating rates. Another aspect that stood out in the DTA curves was the stabilization disorder at the initial temperature of degradation. The more clearly occurring reaction initiation mechanism for the PMMA-6 sample was found to be more stable for the PMMA-12 sample. The cause of this instability, which occurred independently of the heating rate, was the effect of supplementary nanoparticles on the polymer structure. As the metal oxide was concentrated in certain regions (as a result of accumulation) and did not disperse evenly across the entire composite, it caused different thermal effects within the composite under the heat effect and likely caused distortion in such a way that affected subsequent processes. It is inevitable that this process will cause slight mass loss. This agglomeration is the cause of the deviations in the curves obtained from the samples under different heating rates. The data calculated as a result of the DTA are shown in Table 1.

An examination of the T_x_ values as a function of the heating rate shows that the peak temperature increases with increasing heating rates. For the PMMA sample, the T_x_ values were observed to be 651, 661, 670, and 676 K at heating rates of 5, 10, 15, and 20 K/min, respectively. A similar trend was observed for the PMMA-6 and PMMA-12 nanocomposite samples.

### 3.4. Kinetic Analysis of Thermal Degradation

Thermal analysis techniques are widely used to investigate the thermal behavior and degradation characteristics of polymer-based materials. These techniques provide important information about heat flow, phase transitions, and degradation processes occurring in materials during heating. In non-isothermal thermal analysis, the heating rate (β = dT/dt) is typically kept constant during the experiment. The shift in the degradation peak temperatures observed at different heating rates can be used to determine the apparent activation energy of the degradation process.

In the present study, the activation energy of thermal degradation for PMMA and PMMA/Fe_2_O_3_ nanocomposites was calculated using three commonly used non-isothermal kinetic methods: the Kissinger, Takhor, and Augis–Bennett methods.

According to the Kissinger method, the activation energy can be calculated from the following equation:

ln(β/T_x_^2^) = −E_a_/(R T_x_) + C (1)

where β is the heating rate (K/min), T_x_ is the absolute temperature corresponding to the maximum degradation peak, E_a_ is the activation energy, R is the universal gas constant (8.314 J mol^−1^ K^−1^), and C is a constant. The activation energy can be determined from the slope of the linear plot of ln(β/T_x_^2^) versus 1/T_x_.

Another widely used method for activation energy calculation is the Takhor method, which is expressed as

ln(β) = −E_a_/(R T_x_) + C (2)

In this method, the activation energy is obtained from the slope of the linear plot of ln(β) versus 1/T_x_.

The Augis–Bennett method also allows the estimation of the activation energy and is given by

ln[β/(T_x_ − T_0_)] = −E_a_/(R T_x_) + C (3)

where T_0_ represents the onset temperature of the degradation peak. As in the other methods, the activation energy is determined from the slope of the linear relationship between ln[β/(T_x_ − T_0_)] and 1/T_x_.

### 3.5. Thermal Activation Energies

The activation energies of thermal degradation were estimated using the characteristic peak temperatures obtained from the DTA curves at different heating rates. The kinetic parameters were evaluated using the Kissinger, Takhor, and Augis–Bennett methods. These approaches are widely used for evaluating apparent activation energies from non-isothermal thermal analysis data derived from polymeric systems [29,30,31,32].

**Figure 5:**
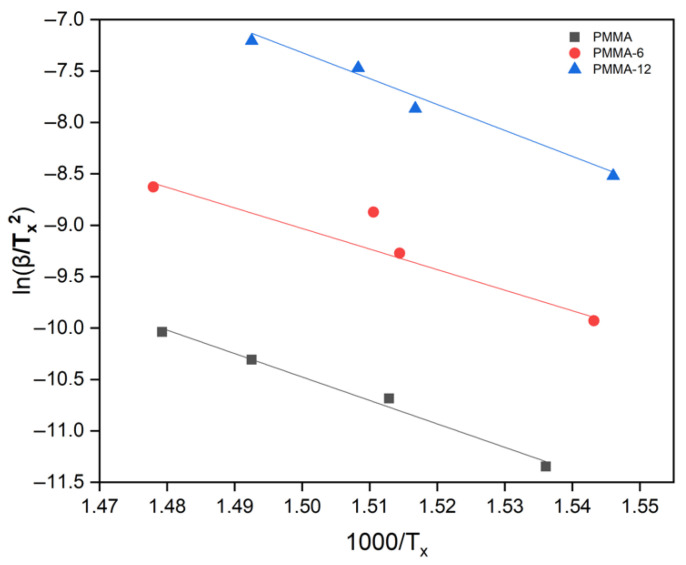
ln(β/T_x_^2^) versus 1000/T_x_ plot for PMMA (pure PMMA used as reference material in this study; adapted from [16], MDPI, 2025), PMMA-6, and PMMA-12 samples according to Kissinger formula.

The Kissinger method is one of the most commonly used techniques to determine the activation energy of thermal degradation reactions in polymers. The relationship between ln(β/T_x_^2^) and 1000/T_x_ for the PMMA, PMMA-6, and PMMA-12 samples is shown in Figure 5, where β represents the heating rate and T_x_ corresponds to the maximum mass loss temperature [30]. From the slopes of the linear plots, the activation energies were calculated as 189 kJ/mol for PMMA, 166 kJ/mol for PMMA-6, and 209 kJ/mol for PMMA-12. The lower activation energy observed for the PMMA-6 sample may be attributed to the insufficient dispersion of Fe_2_O_3_ nanoparticles and the presence of agglomerated regions within the polymer matrix. In contrast, the higher activation energy obtained for the PMMA-12 sample indicates that improved nanoparticle dispersion increases the resistance of the nanocomposite to the main degradation process. The values of the experimental results are presented in Table 2.

**Figure 6:**
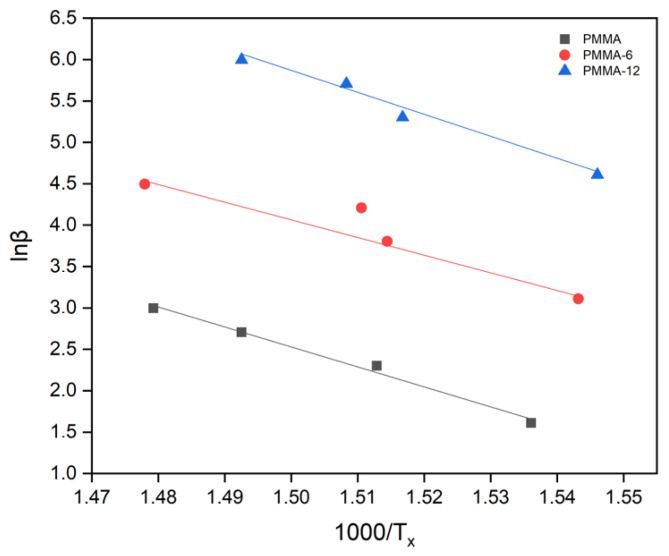
lnβ − 1000/T_x_ graph for PMMA (pure PMMA used as reference material in this study; adapted from [16], MDPI, 2025), PMMA-6, and PMMA-12 samples according to Takhor formula.

Increasing the mixing time results in the improved thermal stabilization of the composite. It is seen that thermal stabilization increases with an increase in the homogenization of the mixing process. When the improved dispersion of the nanoparticles is achieved within the PMMA matrix, the distance between the particles decreases, forming tight binding. Thus, the produced nanocomposite becomes denser and more stable. Therefore, the transfer of heat from one surface to the other surface of the related nanocomposite film becomes easy. In this case, it is possible to form thermally more efficient conductive pathways [26,31]. The activation energies were also evaluated using the Takhor method. The corresponding plots of ln(β) versus 1000/T_x_ derived from the DTA measurements are presented in Figure 6. According to this method, the activation energies were calculated as 200 kJ/mol for PMMA, 177 kJ/mol for PMMA-6, and 220 kJ/mol for PMMA-12.

The results obtained from the Takhor analysis are consistent with those obtained from the Kissinger method and indicate that the PMMA-12 sample exhibits improved thermal stability compared with the other samples. Similar approaches have been used to investigate the degradation behavior of polymer nanocomposites reinforced with inorganic nanoparticles [26,33].

**Figure 7:**
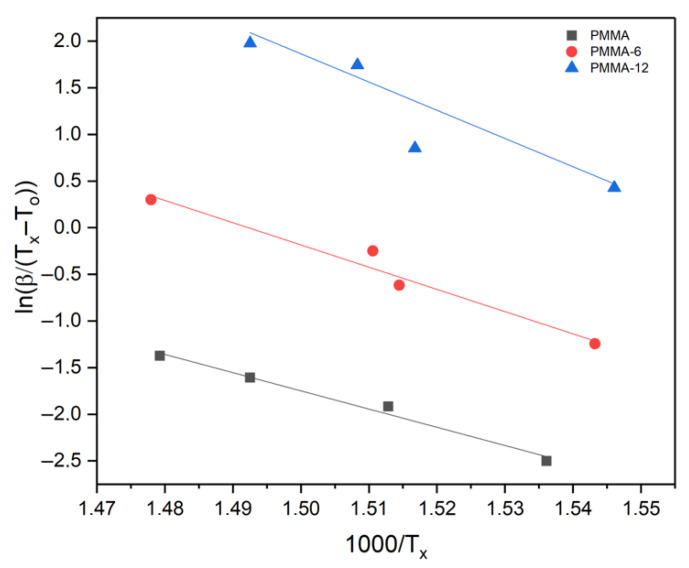
ln(β/(T_x_ − To)) − 1000/T_x_ chart for samples according to Augis–Bennett formula. The PMMA reference data are adapted from [16], MDPI, 2025.

In order to further confirm the kinetic behavior, the activation energies were also determined using the Augis–Bennett method. The plots of ln(β/(T_x_ − T_0_)) versus 1000/T_x_ are shown in Figure 7. The calculated activation energies were 162 kJ/mol for PMMA, 198 kJ/mol for PMMA-6, and 251 kJ/mol for PMMA-12. Although the numerical values obtained from the three kinetic approaches differ slightly due to the assumptions inherent in each method, all three analyses consistently indicate that the PMMA-12 sample exhibits the highest apparent activation energy.

This behavior can be attributed to improved nanoparticle dispersion at longer mixing times. Better dispersion increases the interfacial contact area between Fe_2_O_3_ nanoparticles and PMMA chains, which can influence the degradation mechanism of the composite. Similar effects of nanoparticle dispersion on degradation kinetics have been reported in previous studies on oxide-reinforced polymer nanocomposites [3,33,34].

The activation energy values obtained in this study fall within the ranges reported in the literature for PMMA-based nanocomposites containing inorganic fillers. For example, Ayanoğlu and Doğan reported activation energies of approximately 198 kJ/mol for PMMA/MWCNT nanocomposites using non-isothermal kinetic analysis [26]. Likewise, studies on PMMA systems reinforced with metal oxide nanoparticles have shown that the incorporation of inorganic particles can significantly modify the degradation kinetics and thermal stability of the polymer matrix [3,33,34].

Therefore, the results obtained in the present study confirm that Fe_2_O_3_ nanoparticles influence the degradation behavior of PMMA in a manner consistent with other oxide-reinforced PMMA nanocomposite systems reported in the literature. The results of the calculations performed with the Kissinger, Takhor, and Augis–Bennett methods were similar. The values calculated by these methods are given in Table 2.

The differences among the activation energy values obtained from the Kissinger, Takhor, and Augis–Bennett methods arise from the different mathematical assumptions and thermal parameters used in these approaches. In particular, the Augis–Bennett method includes the onset temperature (T_0_), making it more sensitive to the early stage of degradation, whereas the Kissinger and Takhor methods are more strongly influenced by the temperature of the maximum degradation peak (T_x_). Therefore, differences in dispersion quality and local heterogeneity may affect these methods in different ways, as seen in particular for the PMMA-6 sample.

### 3.6. Thermogravimetric Analysis

**Figure 8:**
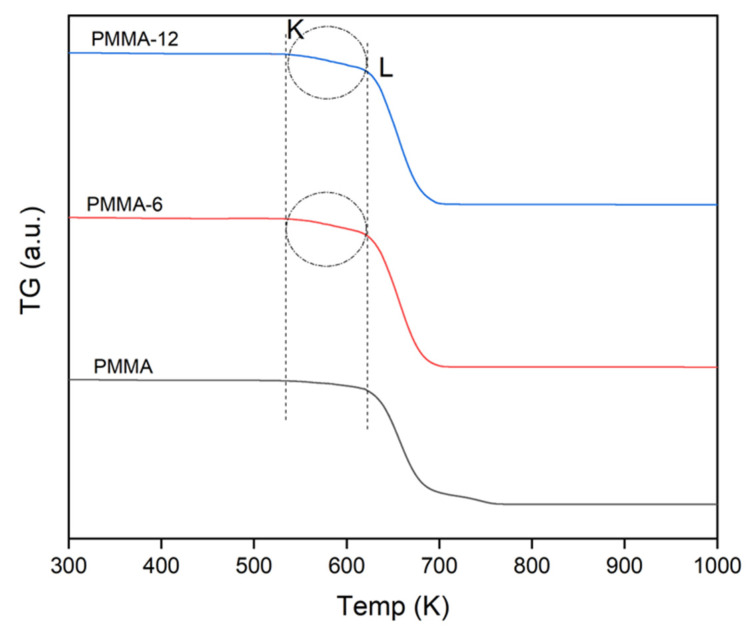
Thermogravimetric (TG) curves of PMMA, PMMA-6, and PMMA-12 samples obtained at a heating rate of 10 K/min, showing the mass loss behavior during thermal degradation.

Thermogravimetric (TG) analysis was performed in order to evaluate the degradation behavior of PMMA and the Fe_2_O_3_-reinforced PMMA nanocomposites. The TG curves obtained at a heating rate of 10 K/min are presented in Figure 8. The pure PMMA sample exhibits the earliest onset of degradation and a sharper mass loss profile. In contrast, the PMMA-6 and PMMA-12 samples show improved thermal stability, indicating that the incorporation of Fe_2_O_3_ nanoparticles delays the degradation process. The PMMA-12 sample demonstrates the highest thermal stability, which can be attributed to the improved dispersion of Fe_2_O_3_ nanoparticles within the PMMA matrix. Better particle dispersion increases the interfacial interaction between the polymer chains and nanoparticles, restricting polymer chain mobility and thereby enhancing thermal resistance.

The degradation parameters, including T_5_, T_30_, T_50_, T_80_, and T_x_, were calculated from the thermograms and are summarized in Table 3. In addition, the parameters K and L represent the remaining sample mass values at specific regions of the TG curve used for evaluating the degradation behavior. The TG results show that pure PMMA undergoes a characteristic thermal degradation process with a maximum degradation temperature (T_x_) of approximately 661 K. After the addition of Fe_2_O_3_ nanoparticles, slight decreases were observed in the characteristic degradation temperatures of the nanocomposites. For example, the maximum degradation temperatures were determined as 660 K for PMMA-6 and 659 K for PMMA-12. Similar trends were observed for the other characteristic temperatures, such as T_5_, T_30_, T_50_, and T_80_. The decrease in the degradation temperature after Fe_2_O_3_ addition can be attributed to the interaction between Fe_2_O_3_ nanoparticles and PMMA polymer chains. The presence of inorganic nanoparticles may introduce local heterogeneities within the polymer matrix and create regions that facilitate earlier chain scission during thermal degradation. Similar effects have been reported for polymer nanocomposites containing metal oxide nanoparticles, where the addition of inorganic particles modifies the degradation pathway of the polymer matrix [33,35]. It should be emphasized that the Fe_2_O_3_ content was kept constant in both nanocomposite samples, and the main experimental variable in this study was the mixing time. Therefore, the observed differences in thermal degradation behavior are primarily associated with the dispersion quality rather than the filler concentration. The slight reduction in some degradation temperatures after Fe_2_O_3_ addition may be related to local heterogeneities and earlier chain scission at the polymer–particle interface, whereas the increase in the apparent activation energy at longer mixing times reflects the influence of improved dispersion on the overall degradation process.

Another important factor affecting the thermal degradation behavior is the mixing time used during the melt-blending process. The SEM observations indicated that the PMMA-6 sample contained noticeable nanoparticle agglomerates, whereas the PMMA-12 sample exhibited a more homogeneous distribution of Fe_2_O_3_ nanoparticles. Increasing the mixing time significantly improved nanoparticle dispersion and reduced agglomeration within the polymer matrix. Improved nanoparticle dispersion increases the interfacial contact area between the nanoparticles and polymer chains, which can influence heat transfer behavior and degradation kinetics within the nanocomposite. As a result, the PMMA-12 sample exhibited a slightly lower degradation temperature but higher apparent activation energy compared with the PMMA-6 sample.

The increase in activation energy with improved dispersion suggests that more homogeneous nanoparticle distribution creates a more stable degradation environment, even though the initial degradation may start at slightly lower temperatures. This behavior has also been observed in other polymer nanocomposite systems, where improved filler dispersion modifies the degradation mechanism and kinetic parameters of the polymer matrix [35]. Similar effects of magnetic particle dispersion on the structural and physical behavior of polymer-based systems have also been reported in magnetically responsive polymer materials, where the aggregation states of magnetic particles strongly influence the overall material properties [36]. In addition, the TG results show that the residue amount increases with the addition of Fe_2_O_3_ nanoparticles. While the residue amount of pure PMMA is approximately 0.42%, the residue values increase to 0.90% for PMMA-6 and 1.43% for PMMA-12. This increase is expected because Fe_2_O_3_ nanoparticles remain as inorganic residues after the degradation of the organic polymer matrix. Aygahoglu et al. investigated the thermal conductivity of iron oxide powders at low temperatures and found that the thermal conductivity coefficients of the samples were affected by the temperature change, depending on the content and size of the Fe_2_O_3_ particles [37]. Overall, the TG analysis confirms that the addition of Fe_2_O_3_ nanoparticles and the mixing time used during melt processing both influence the thermal degradation behavior of PMMA nanocomposites.

It should also be noted that the observed shifts in the characteristic degradation temperatures are relatively small; therefore, they should be interpreted as modest changes in thermal behavior rather than drastic differences in thermal stability.

TG represents the initial sample mass used in the thermogravimetric measurement, while K and L represent the remaining sample mass values at specific regions of the TG curve used for evaluating degradation behavior. This result indicates that PMMA degradation is accelerated by the presence of Fe_2_O_3_ nanoparticles. The reason for the acceleration is that Fe_2_O_3_ nanoparticles may disturb the local chain organization of the polymer structure. Fe_2_O_3_ nanoparticles restrict the mobility of PMMA chains through interfacial interactions due to the fact that polymer chains are connected to inorganic particles. A small amount of iron oxide can reportedly create such acceleration in the thermal decomposition of organics [38].

### 3.7. Application Prospects

The results obtained in this study suggest that Fe_2_O_3_-reinforced PMMA nanocomposites exhibit modified thermal degradation behavior compared with pure PMMA. The presence of Fe_2_O_3_ nanoparticles influences the thermal stability and degradation kinetics of the polymer matrix through interfacial interactions and nanoparticle dispersion effects.

These nanocomposites may be considered for applications where controlled thermal behavior and improved filler–matrix interaction are desirable. Potential application areas include protective polymer coatings, electronic encapsulation materials, lightweight composite components, and polymer-based functional layers used in thermal management systems.

Furthermore, the ability to control nanoparticle dispersion through processing parameters such as the mixing time provides an additional tool for tailoring the thermal behavior of PMMA-based nanocomposites.

Future research should focus on optimizing nanoparticle loading levels, improving dispersion strategies, and investigating additional functional properties such as mechanical strength, dielectric behavior, and long-term thermal stability. The investigation of other metal oxide nanoparticles or hybrid nanofillers may also enable further improvements in the performance of PMMA-based nanocomposites.

## 4. Conclusions

In this study, Fe_2_O_3_-reinforced PMMA nanocomposites were successfully prepared by melt blending using a twin-screw micro-extruder. The influence of nanoparticle addition and the mixing time on the structural, morphological, and thermal degradation behavior of the nanocomposites was systematically investigated. XRD analysis confirmed that the PMMA matrix retained its amorphous structure after the incorporation of Fe_2_O_3_ nanoparticles. Diffraction peaks corresponding to Fe_2_O_3_ were also detected, indicating the presence of the oxide phase within the polymer matrix. SEM observations revealed that the dispersion state of Fe_2_O_3_ nanoparticles strongly depends on the mixing time used during processing. The PMMA-6 sample showed noticeable nanoparticle agglomeration, whereas the PMMA-12 sample exhibited a more homogeneous nanoparticle distribution within the polymer matrix.

The thermal analysis results demonstrated that the addition of Fe_2_O_3_ nanoparticles slightly reduced the characteristic degradation temperatures of PMMA. However, the apparent activation energies calculated using the Kissinger, Takhor, and Augis–Bennett methods increased for the PMMA-12 sample, indicating improved resistance to the main degradation process when nanoparticle dispersion was enhanced. The results obtained in this work show that nanoparticle dispersion and interfacial interactions play a significant role in determining the thermal degradation behavior of PMMA/Fe_2_O_3_ nanocomposites. The findings provide useful insights into the relationship between processing conditions, nanoparticle dispersion, and thermal properties in polymer nanocomposite systems.

The results demonstrate that focusing on processing parameters such as the mixing time can be an effective strategy for controlling nanoparticle dispersion and tuning the thermal degradation behavior of PMMA-based nanocomposites.

## Figures and Tables

**Table 1:** The values for the samples calculated as a result of DTA.

Sample	β (K/min)	T_o_ (K)	T_x_ (K)	A
PMMA	5	590	651	266
	10	593	661	279
	15	595	670	311
	20	597	676	281
PMMA-6	5	556	648	372
	10	562	660	323
	15	560	662	323
	20	598	676	289
PMMA-12	5	594	646	337
	10	590	659	306
	15	620	663	274
	20	625	670	249

**Table 2:** Activation energies calculated from the values of the samples as a result of DTA.

Sample	Kissinger Method (kJ/mol)	Takhor Method (kJ/mol)	Augis–Bennett Method (kJ/mol)
PMMA	189	200	162
PMMA-6	166	177	198
PMMA-12	209	220	251

**Table 3:** Mass loss values for PMMA, PMMA-6, and PMMA-12 samples.

Sample	TG (µg)	K (µg)	L (µg)	T_5_ (K)	T_30_ (K)	T_50_ (K)	T_80_ (K)	T_x_	Residue %
PMMA	11.19	11.08	10.26	601	647	658	682	661	0.42
PMMA-6	13.01	12.89	11.45	582	641	653	671	660	0.90
PMMA-12	13.32	13.19	11.68	578	640	653	671	659	1.43

## Data Availability

The original contributions presented in this study are included in the article. Further inquiries can be directed to the corresponding author.

## Abbreviations

The following abbreviations are used in this manuscript:

Symbol	Meaning
APTS	(3-Aminopropyl) triethoxysilane
DTA	Differential thermal analysis
JCPDS	Joint Committee on Powder Diffraction Standards Card No.
K	Amount of substance remaining in K region
L	Amount of substance remaining in L region
M.W.	Weight-average molecular weight
PMMA	Poly(methyl methacrylate)
PMMA-6	Nanocomposite with 2.5% reinforcement particle ratio prepared by mixing for 6 min
PMMA-12	Nanocomposite with 2.5% reinforcement particle ratio prepared by mixing for 12 min
Residue	Remaining substance as percentage
SEM	Scanning electron microscopy
TG	Initial amount of substance
XRD	X-ray diffraction
A	Area under the DTA peak
β	Heating rate
Tx	Maximum degradation peak temperature
T0	Onset temperature of degradation
